# Protic Processes in an Extended Pyrazinacene: The Case of Dihydrotetradecaazaheptacene

**DOI:** 10.3390/molecules29102407

**Published:** 2024-05-20

**Authors:** Aël Cador, Samia Kahlal, Gary J. Richards, Jean-François Halet, Jonathan P. Hill

**Affiliations:** 1 French Alternative Energies and Atomic Energy Commission, CEA Saclay, DRF/IRAMIS/NIMBE/LSDRM, F-91191 Gif-sur-Yvette, France; ael.cador@cea.fr; 2 Ecole Nationale Supérieure de Chimie de Rennes (ENSCR), CNRS, Institut des Sciences Chimiques de Rennes (ISCR), University of Rennes, UMR 6226, 11 Allée de Beaulieu, F-35708 Rennes, France; samia.kahlal@univ-rennes.fr; 3 Department of Applied Chemistry, Graduate School of Engineering and Science, Shibaura Institute of Technology, Fukasaku 307, Minuma-ku, Saitama-shi 337-8570, Saitama, Japan; richards@shibaura-it.ac.jp; 4 CNRS–Saint-Gobain–NIMS, IRL 3629, Laboratory for Innovative Key Materials and Structures (LINK), National Institute for Materials Science (NIMS), Tsukuba 305-0044, Ibaraki, Japan; 5 Research Center for Materials Nanoarchitectonics, National Institute for Materials Science, Namiki 1-1, Tsukuba 305-0044, Ibaraki, Japan

**Keywords:** pyrazinacene, acene, *N*-heteroacene, azaacene, tautomerization

## Abstract

Pyrazinacenes are linearly fused heteroaromatic rings, with N atoms replacing all apical CH moieties. Component rings may exist in a reduced state, having NH groups instead of N, causing cross-conjugation. These compounds have interesting optical and electronic properties, including strong fluorescence in the near-infrared region and photocatalytic properties, leading to diverse possible applications in bio-imaging and organic synthesis, as well as obvious molecular electronic uses. In this study, we investigated the behavior of seven-ring pyrazinacene 2,3,11,12-tetraphenyl-7,16-dihydro-1,4,5,6,7,8,9,12,13,14,15,16,17,18-tetradecaazaheptacene (**Ph_4_H_2_N_14_HEPT**), with an emphasis on protic processes, including oxidation, tautomerism, deprotonation, and protonation, and the species resulting from those processes. We used computational methods to optimize the structures of the different species and generate/compare molecular orbital structures. The aromaticity of the species generated by the different processes was assessed using the nucleus-independent chemical shifts, and trends in the values were associated with the different transformations of the pyrazinacene core. The computational data were compared with experimental data obtained from synthetic samples of the molecule ***t*Bu_8_Ph_4_H_2_N_14_HEPT**.

## 1. Introduction

**Figure 1:**
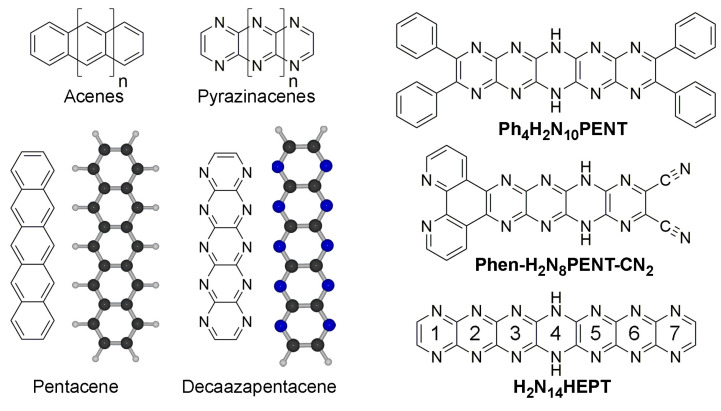
Chemical structures of acenes and pyrazinacenes, including pentacene and 1,4,5,6,7,8,11,12,13,14-decaazapentacene. Energy-minimized structures of pentacene and decaazapentacene are also shown (Atoms: C: black; N: blue; H: white). At right are shown previously prepared examples of pyrazinacenes: **Ph_4_H_2_N_10_PENT** (2,3,9,10-tetraphenyl-6,13-dihydro-1,4,5,6,7,8,11,12,13,14-decaazapentacene [16]), **Phen-H_2_N_8_PENT-CN_2_** (6,13-Dihydrodipyrido[3,2-a:2′,3′-c]-5,6,7,8,11,12,13,14-octaazapentacene-9,10- dicarbonitrile [17]), and, the subject of this work, **H_2_N_14_HEPT** (7,14-dihydro-1,4,5,6,7,8,9,10,13,14,15,16,17,18-tetradecaazaheptacene [18]). Numbering scheme of rings in **Ph_4_H_2_N_14_HEPT** is also shown (see **H_2_N_14_HEPT** in figure).

The term ‘acene’ refers to a series of polycyclic aromatic hydrocarbon compounds composed of linearly fused benzene rings, the archetypal member of which is pentacene [1] (Figure 1), having, as the name suggests, five fused rings. Other members of the series, named according to ring multiplicity, include tetracene [2], hexacene [3], and heptacene [4]. These compounds are investigated for their organic semiconductor properties, although other aspects based on synthetic modifications are available [5,6,7,8]. Longer analogues can behave as one-dimensional conductors complementary to two-dimensional semi-metallic graphene [9]. In terms of real-world applications, acenes have appropriate properties for their use in low-cost large screen displays, or as semiconductor ‘inks’ processable by conventional printing techniques [10].

An unfortunate symptom of increasing ring multiplicity in acenes is a diminishing of the energy gap between the highest occupied molecular orbital (HOMO) and lowest unoccupied molecular orbital (LUMO) and an associated reduction in ionization energy, a feature which is detrimental to their stability against oxidation so that longer acenes (pentacene and beyond) require precautions in their applications or are simply not sufficiently stable [11]. This instability can be accounted for according to Clar’s π–sextet rule since, in acenes, only every other ring can possess the six localized π electrons necessary to fulfil the rule [12]. The number of rings lacking sextets increases in the longer acenes, leading to increasingly poor stability. Recently, there have been several reports of methods for the in situ generation of extended acenes, which avoid synthetic and stability problems, thus allowing for the characterization of these fascinating compounds [13,14,15].

Pyrazinacenes (Figure 1) are *N*-substituted analogues of acenes which contain linearly fused *pyrazine* rings, generally with all acene apical atoms being nitrogen atoms [19,20]. The availability of apical nitrogen atoms allows for the presence of reduced (dihydropyrazine) rings (*sp*^3^ NH instead of *sp*^2^ N) in turn introducing the possibility of multi-stable redox activity or proton delocalization. The compounds can be prepared in a variety of forms, including substituted with solubilizing groups (e.g., **Ph_4_H_2_N_10_PENT** [16], the first synthetically available decaazapentacene; see Figure 1) or with different functionalities (e.g., phenanthrolo-appended **Phen-H_2_N_8_PENT-CN_2_** [17] for metal coordination). Pyrazinacenes can be considered members of the azaacene [21,22] family and are also a subset of heteroacenes [23,24]. Both classes of compounds are investigated for their potential applications as organic semiconductors [25,26], for sensing [27], or other properties [28,29]. Acenes and pyrazinacenes are interesting targets for theoretical studies because of predictions that might be made about properties including their electronic structures [30,31,32] or other aspects of the compounds, including protic tautomerism [33,34].

**Figure 2:**
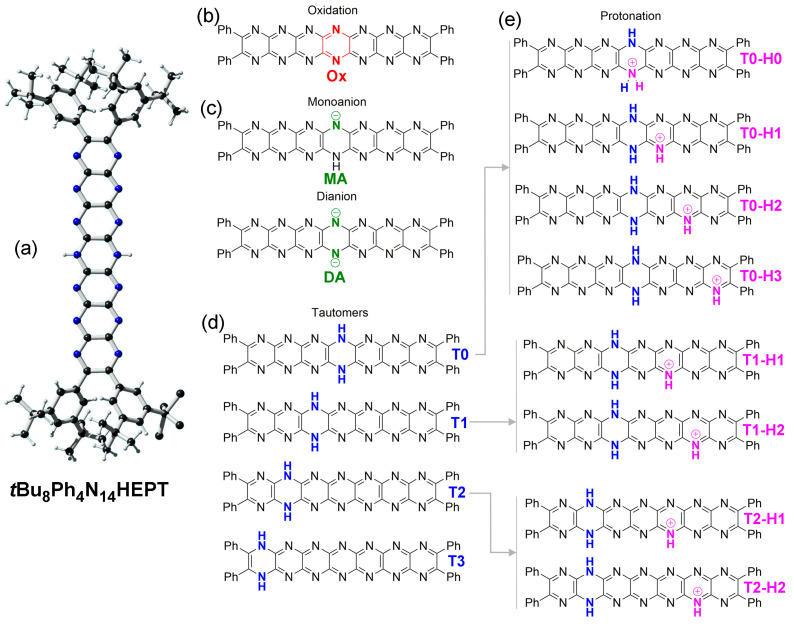
Chemical structures of tetradecaazaheptacenes studied. (**a**) X-ray crystal structure of ***t*Bu_8_Ph_4_H_2_N_14_HEPT,** showing the reduced dihydropyrazine ring located at the central ring [18], (Atoms: C: black; N: blue; H: white). See also Supplementary Materials Figure S1 for a thermal ellipsoid plot. (**b**) The oxidized product, **Ox**. (**c**) Structures of the monoanion, **MA**, and dianion, **DA**. (**d**) Tautomers of **Ph_4_H_2_N_14_HEPT** (**T0**, **T1**, **T2**, and **T3**) available by concerted double proton shifts (other possible tautomers are neglected here). (**e**) Structures of selected protonated tautomers of **Ph_4_H_2_N_14_HEPT**.

In this computational study, the longest of the so-far synthetically available pyrazinacenes [18], namely 7,16-dihydro-1,4,5,6,7,8,9,10,13,14,15,16,17,18-tetradecaazaheptacene (**Ph_4_H_2_N_14_HEPT**, Figure 1 and Figure 2), was considered based on the salient properties of oxidation, protonation/deprotonation, and tautomerism. The compound contains seven linearly fused pyrazine rings with four phenyl substituents at the terminal pyrazine units of the molecule introduced to promote solubility in organic solvents. A single reduced ring is present and is described formally as residing at the center of the molecule as a dihydropyrazine unit. Although we have previously studied extended reduced pyrazinacenes using computational methods [33,34], this work is of interest from the point of view that the chromophore of study was synthesized in previous work [18], enabling a direct comparison of computational data with some of the experimental results. In this case, there exists the possibility of multiple tautomers with the additional aspects of protonation due to the presence of several basic nitrogen atoms, and deprotonation at the dihydropyrazine unit. We have studied the electronic and optical properties of this chromophore based on these transformations using different computational methods.

## 2. Results and Discussion

Here, we focused on several possible transformations of **Ph_4_H_2_N_14_HEPT** in order of their perceived complexity: (i) the simplest possible transformation with the fewest possible products involves two-electron *oxidation* with concurrent elimination of protons at 7,14 positions; (ii) next, we consider *deprotonation*, where stepwise processes lead, respectively, to monoanion and dianion species; and (iii) *tautomerization* is based on isomerization involving variation in the protons’ locations at the different electronegative atoms, i.e., N atoms, of **Ph_4_H_2_N_14_HEPT**. For simplicity here, we consider only isomers where these two protons are shifted to the same adjacent ring so that all isomers contain a dihydropyrazine unit, as shown in Figure 2. Other isomers where the two protons reside on nitrogen atoms of different pyrazine rings contain quinoidal forms, where acene character is lost or interrupted and will be treated elsewhere. (iv) Finally, we consider processes involving *protonation*, and, in this case, monoprotonation is the simplest transformation. Protonation-coupled tautomerized structures are also considered. Each of the aforementioned processes has different connotations for the electronic structure and optical properties of the compound, and we aimed to predict or assess the effects relative to the known properties of this chromophore [18].

### 2.1. Experimental vs. Computed Structures

The X-ray crystal structure of ***t*Bu_8_Ph_4_H_2_N_14_HEPT** (Figure 2a; here, **T0** (**Ph_4_H_2_N_14_HEPT**) is used to differentiate computed (**T0**) from experimental (***t*Bu_8_Ph_4_H_2_N_14_HEPT**) structures) was used as a comparison to test the computational functional/basis set. Excellent agreement with only minor bond length deviations of 0.01–0.02 Å were found between the core chromophores of the two structures (see Table 1).

**Figure 3:**
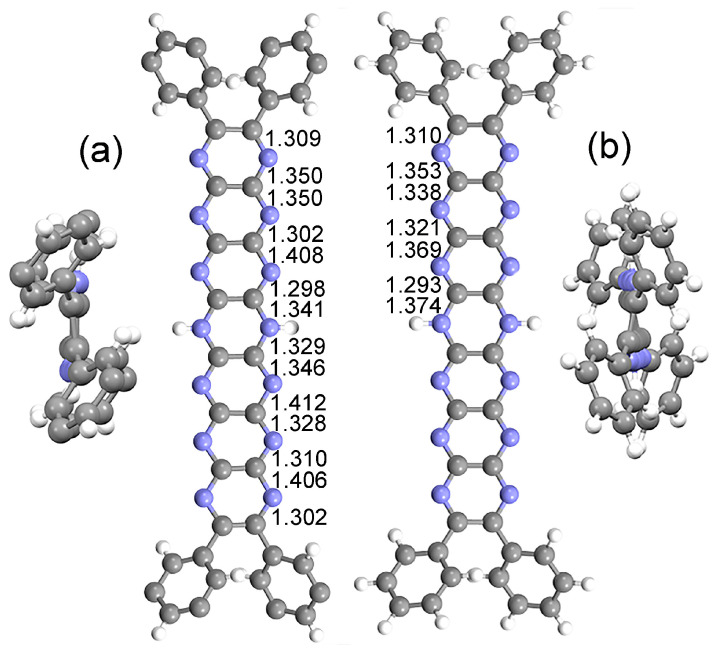
Comparison of experimental (X-ray) and computed structures. (**a**) X-ray crystal structure of ***t*Bu_8_Ph_4_H_2_N_14_HEPT** [18] (*t*Bu groups not shown for clarity) with pertinent C-N bond distances (Å). End-on view (at left) shows the highly planar N_14_-acene core. (**b**) Optimized structure of **T0** (**Ph_4_H_2_N_14_HEPT**) with C-N bond distances (Å). End-on view (at right) shows a (slightly) torsionally distorted acene core.

For ***t*Bu_8_H_2_N_14_HEPT**, its X-ray structure is essentially planar (global torsion angle: 0.7°), while that computed for **T0** (of *D*_2_ symmetry after optimization) has torsional distortion (about 23°), as shown in Figure 3. Both X-ray and computed structures confirm the distribution of the *sp*^3^ character of the dihydropyrazine unit, leading to the planarity of the acene moiety [35]. There is an overall alternation of short and long C-N bond distances in both structures, while the interstitial C-C bond lengths reflect their substantial single bond status. The most significant differences between the two structures involve the N-H bond length, which is shorter than expected for ***t*Bu_8_Ph_4_H_2_N_14_HEPT**, and the dispositions of the phenyl substituents (see Figure 3). The two protons could not be located during refinement (due to poor crystal quality and lower resolution X-ray data), so they were placed using a riding model, which was perturbed during subsequent refinement. Crystal packing forces also affect the form of ***t*Bu_8_Ph_4_H_2_N_14_HEPT** (especially conformation at the phenyl substituents which bear *t*Bu groups), as well as the presence on one 4-dimethylaminopyridine (4-DMAP) molecule hydrogen bonded at each side, making a direct comparison of the two structures less meaningful. However, the main salient features of the molecule (i.e., almost-planar structure, acene-like alternating C-N bond lengths, and strong single bond character in interstitial C-C bonds) found in the experimental structure are reproduced in the computed structure.

### 2.2. Oxidation and Deprotonation

If tautomer **T0** is oxidized involving the loss of two protons/electrons, then the compound labelled **Ox** would be obtained (can also be considered **Ph_4_N_14_HEPT**, labelled **Ox** here for convenience). This compound has a fully conjugated acene-like structure lacking a reduced dihydropyrazine unit (although the reduced ring in those compounds is also subject to delocalization, as has been reported previously [35], and is confirmed here). The HOMO-LUMO gap for **Ox** of 1.813 eV is much lower than that of **T0** (2.499 eV), which is expected for compounds with more highly conjugated electronic structure. Since **Ox** can be formally considered as **T0** that has lost two hydrogen atoms (not protons), the stabilities of its possible singlet and triplet states were assessed. However, it was found that its triplet state is less stable than the singlet state by 14.7 kcal/mol, and the resulting SOMO-LUMO gap is only 0.606 eV.

**Figure 4:**
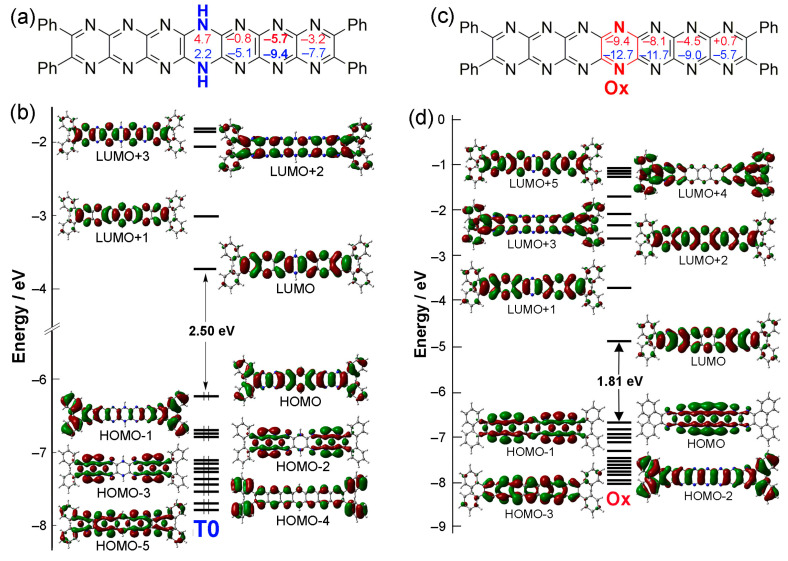
Variations in electronic structures associated with oxidation of **T0** to **Ox**. (**a**) Structure of **T0,** including Nucleus-Independent Chemical Shift (NICS) values (NICS(0), red; and NICS(1), blue). (**b**) MO diagram of **T0**. (**c**) Structure of **Ox**, including NICS values (NICS(0), red; and NICS(1), blue). (**d**) Structures of HOMOs and LUMOs for **Ox**. Contour isodensity values: ±0.02 (e/bohr^3^)^1/2^.

Electronic structures of **T0** and **Ox** are shown in Figure 4. An important feature of the former is the stabilizing distribution of antiaromatic character (introduced by the dihydropyrazine unit; Figure 4a), which yields planar structure despite the *sp*^3^ character of the reduced N atoms [35]. This promotes an acene-like structure in molecular orbitals of **T0** [36], as shown in orbitals HOMO-1 up to LUMO+3 (Figure 4b). HOMO-2 and HOMO-3 indicate a propensity for the delocalization of *N*-atom lone pairs indicated by the overlapping orbital lobes of the hexaazaanthracene groups fused to the central dihydropyrazine. This feature is emphasized in **Ox** (Figure 4c), where HOMO and HOMO-1 (Figure 4d) orbitals exhibit strong lobe overlap between adjacent rings. This aspect of pyrazinacene orbital structure distinguishes them from the CH-acenes; however, the acene character is also retained. The energy order of the orbitals is also quite different: the structures of HOMO-1, HOMO, and LUMO orbitals of **T0** correspond, respectively, to HOMO-2, LUMO, and LUMO+1 orbitals of **Ox**. For the latter, two σ orbitals (HOMO-1 and HOMO) lie between π and π* orbitals, which correspond to the HOMO and LUMO orbitals of **T0**. Thus, the main absorption band of **Ox** can be assigned to a HOMO-2-to-LUMO transition (π → π*) rather than a HOMO-LUMO electronic transition (σ → π*). Table 2 summarizes this situation, where the relevant transition, HOMO-2 to LUMO for compound **Ox**, is also shown to be red-shifted relative to **T0** due to the lower HOMO-LUMO gap of **Ox**.

Nucleus-Independent Chemical Shift (NICS) [37] values are used as a measure of local aromaticity across molecules. Artificial protons positioned at the center of aromatic rings have substantially low chemical shifts, even reaching negative values (−1, −3 ppm…) due to increased shielding; however, this weakens towards the edges due to deshielding. These values can be used to assess aromatic or non-aromatic character at each six-membered ring. The NICS(0) index is the value at the center of the ring, while the NICS(1) index is the value 1 Å above the plane of the ring. Increasingly negative NICS indices indicate higher aromaticity, with increasingly positive values indicating antiaromaticity. For reference here [38], benzene and pyrazine have NICS(0) values of −8.1 and −5.2 ppm, respectively, with values of NICS(1) around −10.0 ppm for both, due to their essential aromatic character. The two conformers of 1,4-dihydropyrazine (boat and chair) have NICS(0) at +11 and +14 ppm, and NICS(1) +8 and +11 ppm, respectively, due to antiaromaticity.

According to NICS indices for **Ox** (Figure 4c), pyrazine-ring aromaticity increases towards the center of the molecule (similar to other acenes [39] and *N*-heteroacenes [40]). In contrast, **T0** has the opposite tendency, with a peak in aromaticity at the center of the conjugated units (note that **T0** can be considered as two hexaazaanthracene units fused through the central dihydropyrazine). In the oxidized compound **Ox**, NICS values are also more homogeneous because of the increasing similarity in character of the fused pyrazine rings of the **H_2_N_14_HEPT** unit and the lack of a reduced dihydropyrazine ring. The terminal pyrazine rings are, however, less similar, as reported previously [33,34].

The presence of the dihydropyrazine ring introduces the possibility of anionic pyrazinacene frameworks by deprotonation. In this case, one or two protons can be successively removed from the dihydropyrazine ring of **T0** [18]. Single deprotonation to the monoanion (**MA** = **[Ph_4_HN_14_HEPT]^–^**) is known to occur easily in the presence of weak bases, such as potassium carbonate, while the formation of the dianion (**DA** = **[Ph_4_N_14_HEPT]^2–^**) is known to require a much stronger base, such as lithium diethylamide (LDA) [16,18]. The effects of these deprotonation processes have been studied here using computational methods. For successive deprotonations of **T0**, the HOMO-LUMO gap decreases from 2.50 eV to 2.06 eV for the monoanion **MA**, and then to 1.75 eV for the dianion **DA**, reflecting some decrease in their stability and/or some increase in their aromaticity.

**Figure 5:**
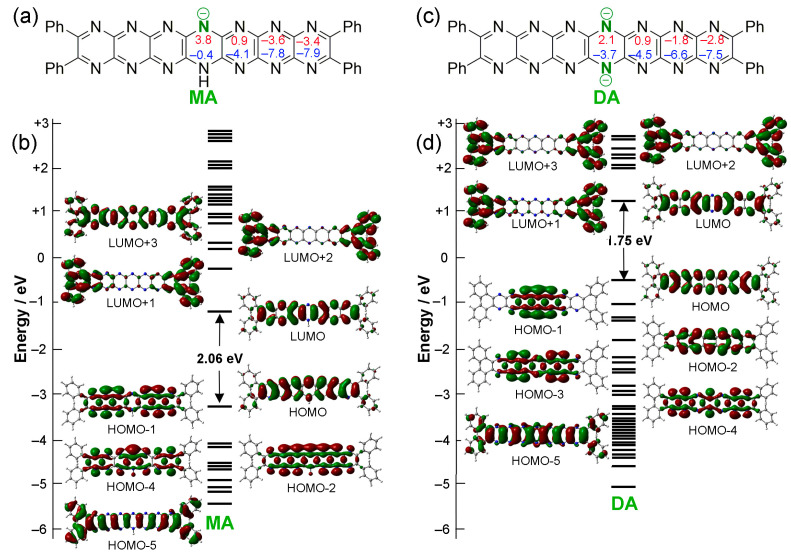
Electronic structures of deprotonated forms of **T0**, monanion (**MA**) and dianion (**DA**). (**a**) Structure of **MA,** including NICS values (NICS(0), red; and NICS(1), blue). (**b**) MO diagram of **MA**. (**c**) Structure of **DA,** including NICS values (NICS(0), red; and NICS(1), blue). (**d**) MO diagram of **DA**. Contour isodensity values: ±0.02 (e/bohr^3^)^1/2^.

Figure 5 shows the formal chemical structures (Figure 5a,c) and molecular orbital diagrams of **MA** and **DA** (Figure 5b,d). The lobes of the molecular orbitals (HOMO-1) lie on the peripheral pyrazine units for **MA**, and on the three central fused pyrazine units for **DA**. For **MA**, its HOMO-2, which is very close in energy to its HOMO-1, also shows contributions at all nitrogen atoms, especially at the central pyrazine unit, while HOMO-4 is highly localized on the central pyrazine ring opposing the remaining proton. The plots of HOMO-1, HOMO-2, and HOMO-4 indicate that contributions from nitrogen lone pairs remain significant after deprotonation with monodeprotonation, reducing the symmetry of the electronic structure. The symmetry is restored by the second deprotonation, with HOMO-1 (now similar in structure to HOMO of **Ox**), HOMO-3, and HOMO-4 showing the impact of the presence of multiple nitrogen atoms.

In this case, the pyrazine groups with the highest NICS aromaticity are found at the molecular ends, remote from the reduced ring, with degree of aromaticity increasing regularly moving outwards from the central dihydropyrazine unit. Deprotonation leads to a stepwise evening of NICS values, most likely as a result of charge being more highly delocalized over all of the rings in both **MA** and **DA**. For this reason, it is not realistic to place localized charges at the central nitrogen atoms of the dihydropyrazine unit (except to denote the chemical structure). The wavelengths of the electronic absorption maxima of the oxidized and dianionic compounds are similar. Initially, this seemed to originate from the similarities in the energies of their HOMO-LUMO gaps and computed structures, which are identical, except for the two extra electrons in the dianionic compound. However, upon closer observation, the structure of **Ox** is subject to more significant torsional distortion than **DA** by about 10°, and its bond lengths do not vary in the same way.

**Figure 6:**
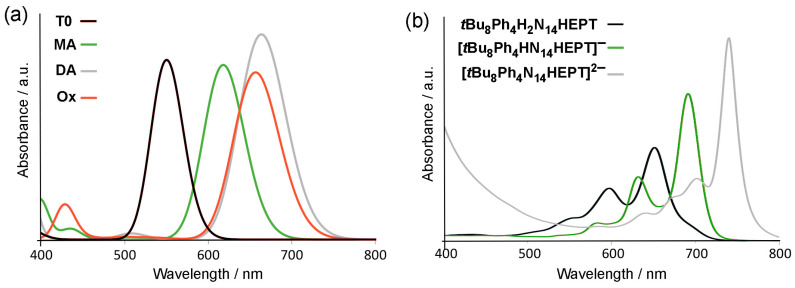
(**a**) Computed electronic absorption (UV-vis) spectra of **T0**, **MA**, **DA**, and **Ox**. (**b**) Experimentally observed UV-vis spectra of **tBu_8_Ph_4_H_2_N_14_HEPT**, **[Ph_4_HN_14_HEPT]^–^**, and **[Ph_4_N_14_HEPT]^2–^** [18]. For solution in (**b**), c = 6 × 10^–6^ M in tetrahydrofuran. Molar extinction coefficient: 116,700 M^–1^ cm^–1^. Stepwise deprotonation was achieved via the addition of lithium bis(trimethylsilyl)amide.

For **MA** and **DA**, the TD-DFT results indicate that the absorption maxima are almost exclusively due to HOMO-LUMO (π → π*) transitions, explaining why the energy of the main absorption wavelength is close to the HOMO-LUMO gaps of each compound (see Table 3). Each deprotonation step is associated with a red shift from 550 (**T0**) to 618 nm (**MA**), and then 618 to 664 nm (**DA**), with corresponding shifts in fluorescence emission (see Figure 6a). This can be compared directly with the experimentally known behavior of ***t*Bu_8_Ph_4_H_2_N_14_HEPT** during deprotonation, using weak and strong bases, where, respective, shifts from 653 to 700 nm then 700 to 741 nm (in THF) have been observed (Figure 6b) [18]. Thus, the TD-DFT results are consistent with the experimental trends. Note that the vibrationally resolved electronic absorption spectra were not computed to confirm the vibronically coupled monotonically diminishing higher energy bands commonly observed in acenes, pyrazinacenes, and other chromophores [41]. Finally, regarding **Ox**, the main electronic absorption earlier assigned to a HOMO-2-to-LUMO transition (π → π*) occurs at 657 nm, representing a 107 nm red shift and a substantial stabilizing effect. However, ***t*Bu_8_Ph_4_H_2_N_14_HEPT** cannot be obtained in the two-electron oxidized state, so a direct comparison of this value is not available. Despite this, in analogous dihydrooctaazatetracene (**H_2_N_8_TET**) and dihydrodecaazapentacene (**H_2_N_10_PENT**) derivatives, oxidation to their respective **N_8_TET** and **N_10_PENT** forms is accompanied by 90 nm red shifts in their absorption maxima. It is not clearly understood why ***t*Bu_8_Ph_4_N_14_HEPT** is not stable; however, reactivity towards trace water is strongly suspected as the reason for this observation. The strongly basic oxidized state ***t*Bu_8_Ph_4_N_14_HEPT** might easily extract a proton from water, facilitating the reduction of the resulting protonated state. It is also possible that commonly available oxidants (e.g., PbO_2_ or high potential quinones such as 2,3-dichloro-5,6-dicyano-1,4-benzoquinone (DDQ)) are simply capable of oxidizing the already electron-deficient ***t*Bu_8_Ph_4_H_2_N_14_HEPT** chromophore. We are currently investigating the role of water in the respective reactions based on the possibility of using extended pyrazinacenes as electro- or photoredox catalysts.

### 2.3. Tautomerization

**Figure 7:**
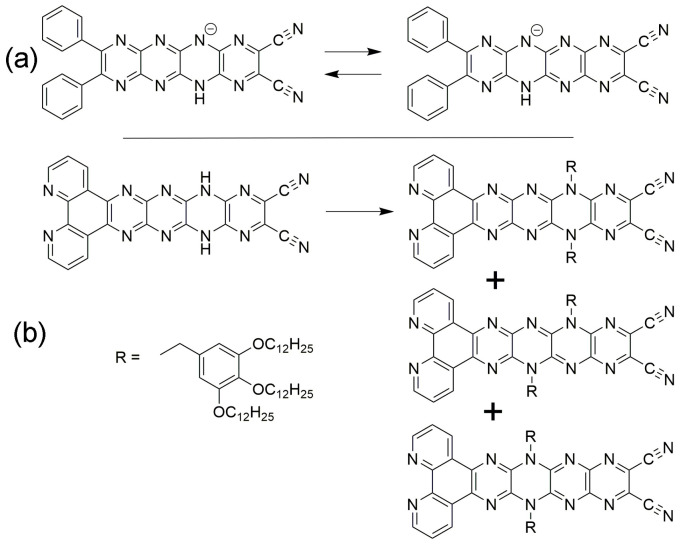
Experimentally observable examples of tautomerism in pyrazinacenes. (**a**) Proton shift in the monoanion of **Ph_2_H_2_N_8_TET-CN_2_** observed directly using low-temperature ^1^H nuclear magnetic resonance [34]. (**b**) *N*-alkylation of a tautomer mixture leads to indirect observation of tautomers in **Phen-H_2_N_8_PENT-CN_2_** [17]. These examples also reveal the inaccessibility of the terminal pyrazine groups in pyrazinacenes, as also found here computationally.

Tautomerization is potentially the most significant of the possible properties of **H_2_N_14_HEPT** derivatives because of implications for intramolecular proton transport or hydrogen-bonding guest adaptability [42]. However, it has so far proved to be the least accessible for study experimentally due to the broadness of the relevant signals in NMR or FTIR spectroscopy; however, tautomerization in an *N*_8_-tetracene monoanion where two tautomers can coexist has been reported [34]. The availability of the tautomers of pyrazinacenes has also been established indirectly by *N*-alkylation of *N*_8_-pentacene derivatives [17]. Examples of these two special observable cases of tautomerization are shown in Figure 7. The different possible dihydropyrazine-containing tautomers of **Ph_4_H_2_N_14_HEPT** (**T0**, **T1**, **T2**, and **T3**) are shown in Figure 2d. Based on experimental observations, tautomerization is favored only in *N*_8_-tetracenes and above because the existence of the dihydropyrazine unit at extremities of the pyrazinacenes is strongly energetically disfavored most likely due to the weaker delocalization of antiaromaticity and the loss of a Clar sextet. Thus, *N*_6_-anthracenes exist as a single tautomer, while symmetrically substituted *N*_8_-tetracenes may exist as two tautomers.

The energies of the different **T0**–**T3** were computed and are compared in Table 4. The tautomers become increasingly unstable as the dihydropyrazine unit shifts away from the center of the molecule, with Δ*E* and Δ*G* following a similar pattern. As expected from experiment, **T3** is significantly less stable than **T1** and **T2**, accounting for the absence of its *N*-alkylates in experiments designed to trap the tautomers by derivatization at the nitrogen atoms [17,34]. The HOMO-LUMO gap (see Table 4) is reduced, being associated with the increasing extent of the conjugated system (three, four, five, and then six rings), since, in general, larger conjugated systems exhibit smaller HOMO-LUMO gaps. Related to this are bond lengths in the tautomers (see the coordinate files of the relevant compounds in Supplementary Materials), where C-C bonds at the more highly conjugated side of the compound are lengthened, while those at the opposing side (i.e., lower conjugation) become shorter (ca. 0.02–0.03 Å). Conversely, the C-N bonds at the more highly conjugated side are shortened significantly (by 0.06–0.08 Å). These variations are consistent with the increasing acene character of the increasingly conjugated moiety and low-acene character of the opposing side, despite the delocalization of the dihydropyrazine unit.

In **T0** (see Figure 4a for the NICS values), NICS values indicate a reduction in the antiaromatic character of the dihydropyrazine ring relative to the parent 1,4-dihydropyrazine, while the largest aromaticity is located at a point generally as far from the dihydropyrazine unit as possible without being on the terminal pyrazine group. For regular acenes and azaacenes, aromaticity is generally the greatest at the center of the molecules. In this case, NICS values indicate that the antiaromatic character of the 1,4-dihydropyrazine ring is distributed over the molecule and should be associated with the planarity of the molecule. This has been reported previously in the case of dihydro-6,13-diazapentacene [35], although the multiplicity of pyrazine units in **T0** led us to confirm this situation. Incidentally, the NICS values of the phenyl groups are similar to those of benzene despite some orbital participation indicated by the frontier molecular orbital structures of **T0** (Figure 4b, HOMO and HOMO-1).

For **T0**–**T3** compounds containing 14 N atoms and a single dihydropyrazine unit, the sites at which protons of the reduced ring reside is an important parameter especially in the context of molecular recognition events, where proton location would affect intermolecular interactions. Local charge or orbital structure might affect the location of those protons. To investigate this point, natural charges were estimated using natural bond orbital (NBO) analysis, revealing that the most highly charged atoms are those of the dihydropyrazine ring, with others being slightly less negative. Notably, terminal pyrazine rings 1 and 7 show a persistent charge of –0.37, which hardly varies except when adjacent to the dihydropyrazine unit.

**Figure 8:**
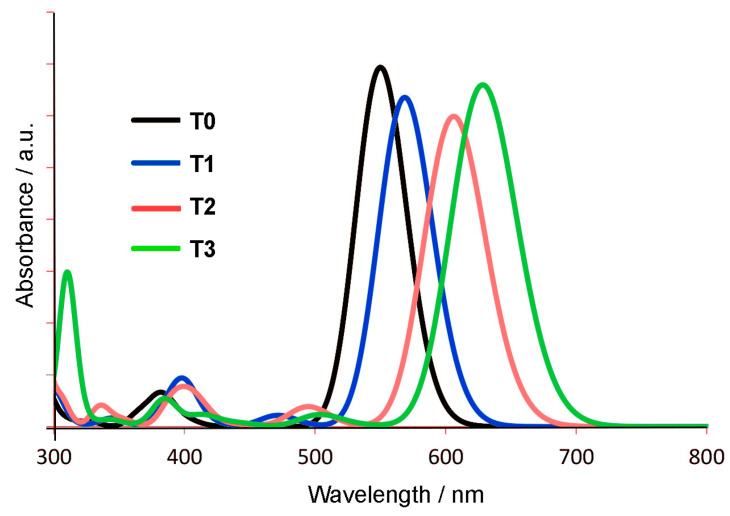
Calculated electronic absorption spectra for the tautomers **T0**–**T3**. Note the stepwise red shift.

For electronic absorption spectra (UV-vis), the lowest energy computed wavelength of the absorption maximum is due largely to a HOMO-LUMO electronic transition, and there is a successive 20–30 nm red shift in the absorption band for the less stable tautomers (see Table 5). This should be accompanied by a significant Stokes shift (from 35 to 85 nm) of the fluorescence emission band. The calculated UV-vis spectra for **T0**–**T3** are shown in Figure 8. In this case, the red shifts in absorption maxima can be assigned to the increasing extent of the delocalized π electronic system, which has a stabilizing effect. It will be difficult experimentally to confirm these values due to the large number of possible tautomers available for **Ph_4_H_2_N_14_HEPT** (13 possible tautomers in symmetrical derivatives if *N*-alkylation at rings 1 and 7 is neglected). To obtain pure samples of derivatized tautomers will require extensive chromatographic separation and an informed selection of the derivatizing agent. However, this method can be used to fix electronic structures of the N_14_ chromophore, allowing us access to different aromatic and quinoidal forms. We are currently investigating this aspect of the pyrazinacene systems.

### 2.4. Protonation

**Figure 9:**
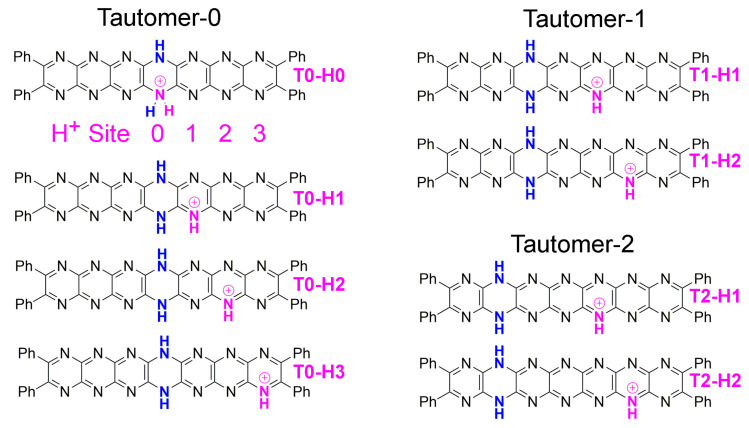
The three series of monoprotonated tautomers. (**Left**): Tautomer-0 (**T0**) with numbering scheme for protonation site. (**Right**): (**top**), Tautomer-1 (**T1**); and (**bottom**), Tautomer-2 (**T2**).

Based on the large number of nitrogen atoms in **Ph_4_H_2_N_14_HEPT** compounds, protonation is potentially the most complicated of its protic processes. For this reason, and because these are currently the least accessible of the real-world isomers, we studied only selected monoprotonated isomers. There are many possible structures available if a reduced **Ph_4_H_2_N_14_HEPT** is monoprotonated based on several different possible tautomers (including unsymmetrical ones) and 12 additional possible protonation positions. If the linear symmetry of **Ph_4_H_2_N_14_HEPT** is taken into account and tautomers are constrained to contain a single neutral dihydropyrazine group, there are 25 possible isomers of [**Ph_4_H_3_N_14_HEPT]^+^**. Here, we selected eight of these using the most stable neutral tautomers as starting points. The chemical structures of the protonated tautomers studied here are shown in Figure 9. Each protonated tautomer is identified based on the starting tautomer structure and the protonation site. There are three series of isomers: **T0-HX**, **T1-HX,** and **T2-HX,** where **X** denotes the protonation site of four selected, as indicated below, **T0**-**H0** in Figure 9. Note that protonation on the dihydropyrazine ring was also considered (Site 0); however, this is highly unlikely given the weak *sp*^3^ character of those atoms. Also, **T3** was neglected because of the low stability of that tautomer.

The computed results shown in Table 6 indicate that the stability of the compounds is promoted if protonation is (in order of importance) (i) not at the reduced dihydropyrazine ring, (ii) not at a terminal pyrazine ring, and (iii) separated from the reduced dihydropyrazine by two pyrazine units. Interestingly, the most stable of these compounds (**T1-H2**) is found in the **T1** series rather than in the **T0** series, as might be expected based on the overall greater stability of **T0**. Overall, the stabilities suggest that there is a balance between tautomer stability and the site of protonation for these isomers, and that protonation might be reasonably used to affect tautomer identity. That is, **T0** might rearrange to **T1** upon protonation to optimize stability. In solutions, especially in polar solvents, the situation is likely to be highly complex with protonated tautomers in a fluxional state depending on the acidity and concentration of **Ph_4_H_2_N_14_HEPT**.

HOMO-LUMO gaps of the protonated compounds are lower than their non-protonated counterparts, being the lowest of the compounds discussed here. Essentially, the reduction in gap is inversely connected with the proximity of the protonated ring to the dihydropyrazine unit and is probably related to the relative extent of the non-protonated conjugated system in the different series. In this respect, it should be remembered that the dihydropyrazine ring is effectively delocalized over local non-protonated units. HOMO-LUMO gaps are 0.15–1 eV lower than for the neutral tautomers, and all (except that of **T0-H0**) are lower than that of compound **Ox** (1.81 eV), where all pyrazine rings are conjugated. Protonation causes some small variations in molecular geometry (see the coordinate files of the relevant compounds in the Supplementary Materials), where C-N bonds at the side of the molecule at which protonation occurs are lengthened about the site of the proton and then alternate between shortened and lengthened for each successively remote bond by about 0.02–0.03 Å, up to 0.05 Å. Carbon–carbon bonds shorten somewhat about the site of protonation and lengthen at the remote rings (changes in the range of 0.01–0.02 Å). These changes are likely also connected with variations in local aromaticity. Relative to the parent tautomers, the perturbation of aromaticity due to protonation in most of the isomers increases NICS(1) indices for the pyrazine rings on the protonated side by +2 to +5 ppm in the protonated ring and by +1 to +2 ppm in the others (see next section).

**Figure 10:**
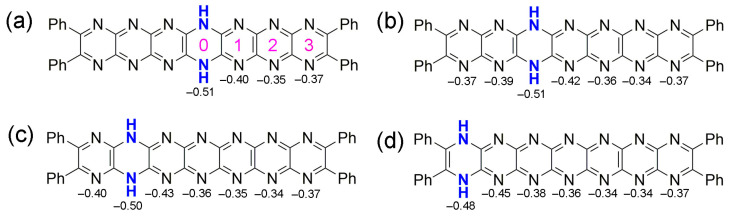
Charges on the rings of the neutral tautomers (**a**) **T0**, (**b**) **T1**, (**c**) **T2**, and (**d**) **T3**. Possible sites of protonation indicated in pink in (**a**).

A consideration of the charges on the neutral compound **T0** might reveal the *N*-atom sites that are preferred for protonation. For **T0**–**T3**, charges at the rings are shown in Figure 10. Values of charge indicate that protonation at Site 0 ought to lead to the most stable isomer, with Site 2 being least favored (a charge value around –0.5 is maintained at the dihydropyrazine ring in all of the tautomers). However, considering the total energies of the protonated compounds, protonation appears to be preferred at less negatively charged sites. For **T0**, protonation can be considered an electrophilic attack and so involves its non-bonding σ molecular orbitals, and the influence of nitrogen lone pairs is important. The MO diagram indicates that HOMO-2 and HOMO-3 have the greatest contribution from the N lone pairs at the first, second, sixth, and seventh rings. For this reason, isomers protonated at the nitrogen atoms of those rings should be the most stable, and this is indeed what is found by calculation—in fact, more so for rings 2 and 6.

**Figure 11:**
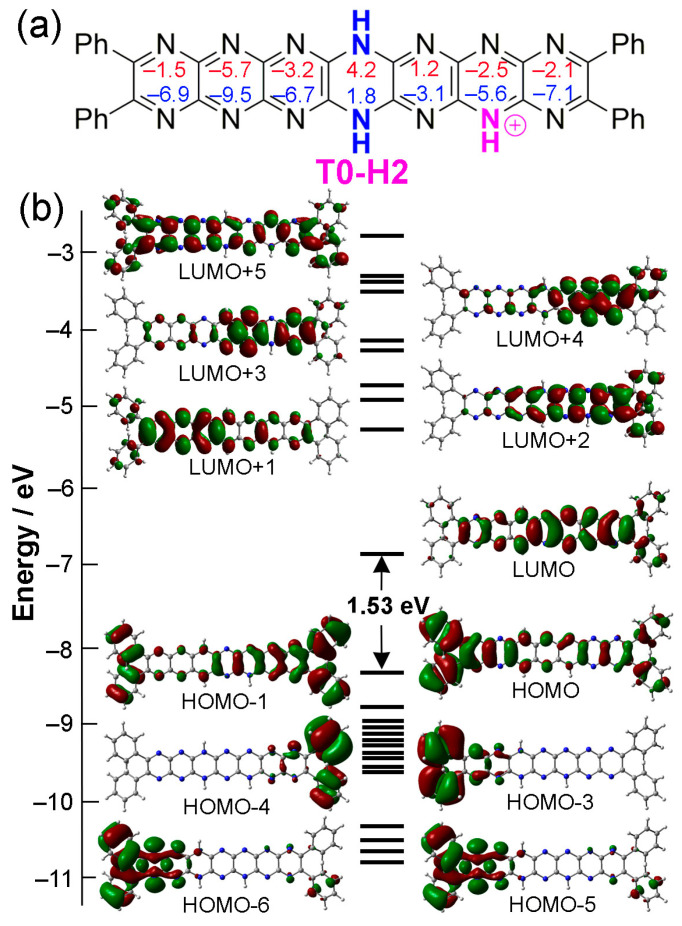
Protonated structure **T0-H2**. (**a**) Structure of **T0-H2**, including NICS values (NICS(0), red; and NICS(1), blue). (**b**) MO diagram of **T0-H2**. Contour isodensity values: ±0.02 (e/bohr^3^)^1/2^.

The isomer **T0-H2**, the most stable in the **T0** family of isomers, was selected to assess the effects of monoprotonation on molecular orbital structure. Figure 11 shows the HOMO and LUMO structures of this isomer. Interestingly, there is almost no evidence for nitrogen lone pair delocalization in **T0**-**H2**, with these features only emerging in deeper HOMO-5 and HOMO-6, where phenyl substituents also contribute. In the HOMO, electron density is situated remotely from the protonation site, while the LUMO is focused about the site. This situation indicates the possibility of electronic push–pull-type behavior or intramolecular electron transfer activity, both of which might be photolytically activated using long-wavelength excitation sources.

**Figure 12:**
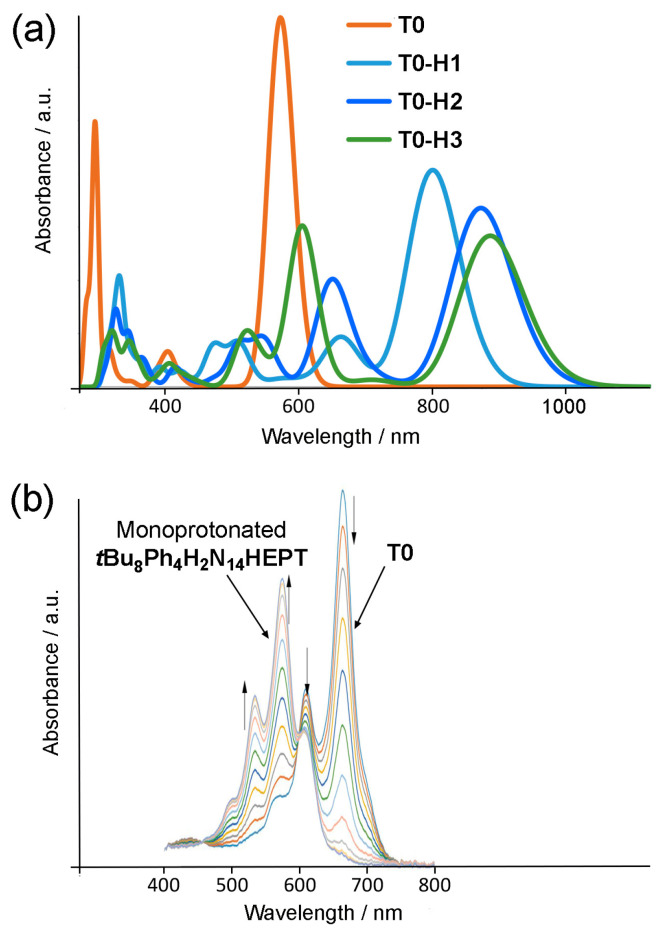
(**a**) Electronic absorption spectra of **T0** and some of its monoprotonated tautomers. Arrows at peak maxima indicate increasing or decreasing absorbance. (**b**) Electronic absorption spectra obtained during titration of ***t*Bu_8_Ph_4_H_2_N_14_HEPT** with trifluoroacetic acid (TFA). For solution in (**b**), c = 6 × 10^–6^ M in tetrahydrofuran. Molar extinction coefficient: 116,700 M^–1^ cm^–1^. Panel (**b**) adapted with permission from ref [18]. Copyright 2019 American Chemical Society.

For **T0**-**H2** and the other stable isomers, the HOMO is quite close in energy (0.36–0.46 eV) to the next occupied orbitals, while the LUMO is separated (by 1 eV or more) from the next vacant molecular orbitals. Hence, electronic excitations must involve HOMO-to-LUMO or HOMO-region-to-LUMO transitions. Similar to the neutral compounds, the HOMOs and LUMOs are of π and π* character, respectively. We indeed observe this type of electronic transition (see Table 7) but exclusively HOMO to LUMO (so the wavelengths follow exactly the same evolution as the gaps), similarly to the neutral compounds. However, since the HOMO-LUMO gaps are smaller, the main absorption wavelengths are much larger, in the near infrared (770–930 nm). The absorption wavelengths increase when the proton is further away from the reduced ring and when the reduced ring is closer to the remote rings, with red shifts between 25 and 80 nm from one series to another. However, the main bands are less intense than those of neutral compounds, and there are other absorptions in the visible region. The intensities also decrease as the wavelengths increase. Computed absorption spectra are shown in Figure 12 for some monoprotonated tautomers of **T0**, while some of the monoprotonated tautomers of **T1** and **T2** are available in Supplementary Materials Figures S2 and S3.

Based on the calculations, both protonation and deprotonation occur with similar red shifts of the absorption maximum; however, this effect is more significant for protonation (for deprotonation, shifts lie in the range between 70 and 115 nm, and for protonation, in the range between 220 and 360 nm). However, while calculations give a reasonable reproduction of the red shifts incurred by stepwise deprotonation (and also for oxidation of **T0**), electronic absorption data collected from acidified solutions of **T0** are not consistent at first sight with the calculated absorption maxima found here. Figure 12 shows a comparison of calculated (Figure 12a) vs. experimental (Figure 12b) electronic absorption spectra of protonated **T0**. The experimentally observed electronic absorption maximum for ***t*Bu_8_Ph_4_H_2_N_14_HEPT** is actually blue-shifted by approximately 100 nm. The reasons for this inconsistency are unclear; however, solvents or impurities such as water are expected to have a significant effect on the stability of protonated tautomers. In the case of deprotonation and oxidation, electron density can be effectively delocalized intramolecularly, minimizing the possible effects of solvation/hydration, while the presence of positive charges after protonation might imply interactions with electronegative solvating agents or water for stabilization.

### 2.5. Trends in Aromaticity Based on NICS

**Figure 13:**
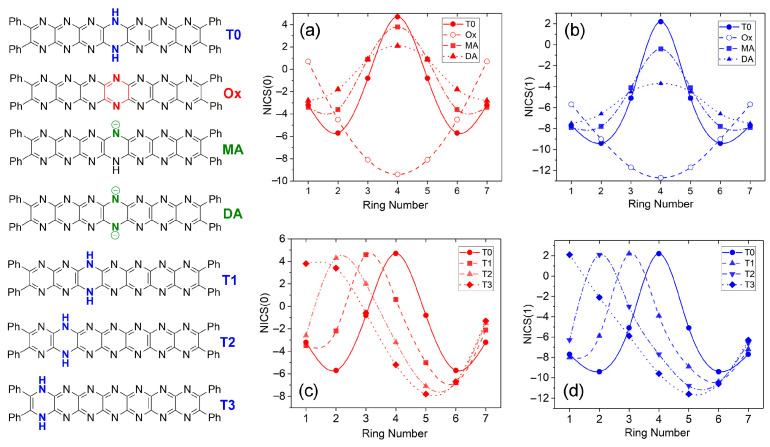
Nucleus-independent chemical shifts (NICS(0,1)) vs. ring number for **T0** (**Ph_4_H_2_N_14_HEPT**), oxidized and anionic species. (**a**) NICS(0) and (**b**) NICS(1) values for **T0**, **Ox**, **MA,** and **DA**. (**c**) NICS(0) and (**d**) NICS(1) values for **T0**–**T3** tautomers.

Perturbation of aromaticity involving the processes studied here can be probed computationally, using NICS indices [37]; the trends can be associated with specific changes in the molecule structures and then visualized. Figure 13 shows variations in NICS(0) (red traces) and NICS(1) (blue traces) indices for **T0**, **Ox**, **MA**, and **DA**. These species are symmetrical about a mirror plane placed perpendicular to the molecules’ long axes at the central ring. Both NICS indices (Figure 13a,b) show symmetric traces based on this. **T0**, **MA**, and **DA** formally contain a dihydropyrazine unit whose antiaromaticity results in a strong positive peak in NICS values at the center of the molecule. For **MA** and **DA**, deprotonation allows for further delocalization of electron density, leading to the distribution of the positive value across all rings and a significant lowering at the central ring. For **Ox**, all pyrazine rings have a substantial aromatic character, and this appears to be focused in intensity at the central pyrazine unit, where there are strongly negative values for both NICS indices. Interestingly, terminal pyrazine rings have significantly less negative NICS values again, emphasizing the different state of these areas of the molecule. For the tautomers **T0**–**T3**, the positive peak in NICS values (see Figure 13c,d) shifts without a substantial change in intensity according to the location of the dihydropyrazine unit, and an increasingly negative peak appears at the central rings (Rings 5, 6) of the widening conjugated region, reflecting the locally increasing aromaticity due to the shift of the dihydropyrazine group.

**Figure 14:**
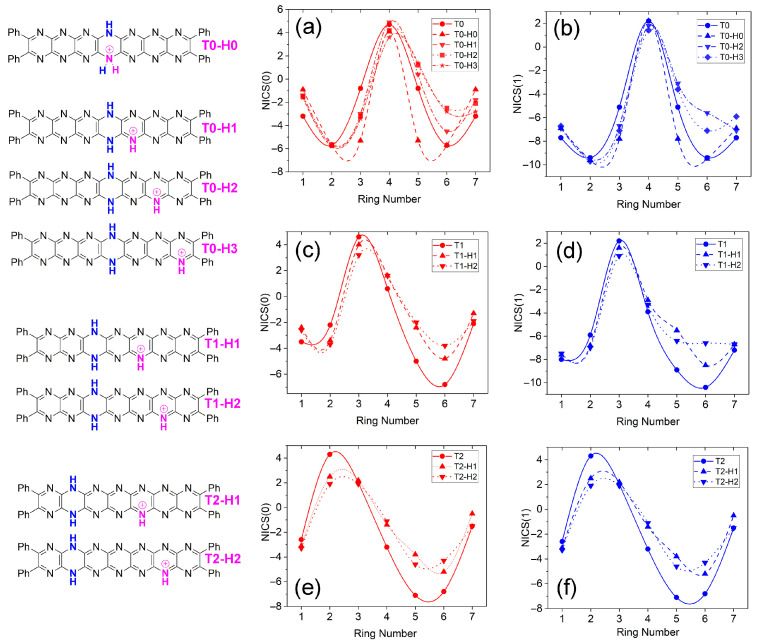
Nucleus-independent chemical shifts (NICS(0 (red), 1 (blue))) vs. ring number for **T0** protonated tautomer isomers. (**a**) NICS(0) and (**b**) NICS(1) values for **T0-H0**–**T0-H3**. (**c**) NICS(0) and (**d**) NICS(1) values for **T1-H1** and **T1-H2** isomers. (**c**) NICS(0) and (**d**) NICS(1) values for **T1**-**H1** and **T1-H2** isomers. (**e**) NICS(0) and (**f**) NICS(1) values for **T2-H1** and **T2-H2** isomers.

Trends in the NICS indices are summarized for protonated tautomers in Figure 14. Similar to the neutral tautomers, the positive peaks in NICS values coincide with the location of the dihydropyrazine ring, and the value magnitude hardly varies. Protonation shifts NICS(0,1) about the location of protonation to less negative values in all cases. In the case of pyridine, NICS values become more negative upon protonation to pyridinium as the electronic structure shifts towards that of benzene, with which it is isoelectronic [37]. Less negative NICS for protonated tautomers of **T0** might reflect an overall shift of electron density away from the site of protonation as suggested to occur the HOMO structures of **T0**-**H2** (Figure 11b), where several of the occupied molecular orbitals reside largely on the phenyl substituents. Alternatively, increasing NICS values occur for the central rings of the conjugated regions of the molecules, perhaps indicating some delocalization of the cationic charge.

## 3. Methods

**Computational Details.** Density functional theory (DFT) was used for geometry optimization of **Ph_4_H_2_N_14_HEPT** [43,44] (and its derivatives), using the *Gaussian 16* program package [45], with the B3LYP functional [46,47,48,49] and the all-electron def2-TZVP basis set of the EMSL Basis Set Exchange Library [50,51,52,53,54]. Geometries optimized without any constraint were characterized as being true minima based on a vibrational analysis. Natural charges were computed using the *NBO 6.0* (now *NBO 7.0*) program [55]. Electronic absorption (UV-vis) spectra were calculated by using time-dependent DFT (TD-DFT) [56,57], at the B3LYP/def2-TZVP level with only singlet-singlet, i.e., spin-allowed, transitions being computed. UV–vis spectra were simulated based on the TD-DFT transitions and their computed oscillator strengths using the *SWizard* program [58]. Each transition was associated with a Gaussian function at half-height width (equal to 1500 cm^−1^). Nucleus-Independent Chemical Shifts (NICSs) were estimated based on NMR calculations at B3LYP/def2-TZVP level [37]. The *AOMix* program was used to calculate the composition of the molecular orbitals [59,60].

## 4. Conclusions

In summary, N_14_heptacene and pyrazinacenes, in general, provide an opportunity to study molecules capable of undergoing all major protic processes within the same chromophore system. This presents unique opportunities for applications in bio-imaging (for instance, local pH mapping) and molecular recognition, including systems involving dynamic proton transport, H-bonding catalysis (including photoredox catalysis), and molecular electronics. In this work, we considered the seven-ring reduced pyrazinacene **Ph_4_H_2_N_14_HEPT**, because its synthetic availability allows for a direct comparison of its properties, especially during protonation and deprotonation. Transformations such as tautomerization and oxidation, although known to occur in lower pyrazinacene analogues, have not been so far observed in **Ph_4_H_2_N_14_HEPT**. While the oxidation of **Ph_4_H_2_N_14_HEPT** is thought to be precluded by its extensive electronic delocalization, **Ox** (**Ph_4_N_14_HEPT**) may also be too reactive to be observed by usual methods. While **Ox** appears to be computationally stable with a smaller HOMO-LUMO gap, as expected for more highly conjugated chromophores, it has an irregular molecular orbital structure, which might also suggest reasons for its instability under normal conditions. This situation can be addressed by observing **H_2_N_14_HEPT** under different conditions, such as adsorbed at being a metal interface [61]. Lower analogues of oxidized pyrazinacenes are known to be stable when adsorbed at Cu(111) surfaces; however, for **H_2_N_14_HEPT**, these measurements are made difficult by its large molecular weight and tendency to decompose when heated at temperatures used for sublimation.

For deprotonation, our computational regime effectively reproduces spectral data, providing a basis for us to investigate the electronic properties of those species. **MA** and **DA** have absorbances and emissions in the near infrared (NIR), making this chromophore highly suitable for bio-imaging applications, possibly as a probe of local pH during intracellular processes. Deprotonation obliterates protic tautomeric processes but introduces other possibilities, such as intramolecular electron transfer, as indicated by the tendency for HOMO and LUMO orbitals to be localized on different regions of the molecule (see Figure 5b,d). For tautomerization of **Ph_4_H_2_N_14_HEPT**, our calculations indicate that the tautomer **T0** having a central dihydropyrazine ring is the most stable, while dihydropyrazine ring shifts away from center lower the HOMO-LUMO gap and red-shift the main UV-vis absorption bands. Amongst the protonated tautomers, the most stable are those where the protonated ring is one or two rings away from the remote rings (e.g., **T0-H2**; see Figure 11). An increase in the proton–hydrogen distance is correlated with a HOMO-LUMO gap decrease, as well as an intensity decrease and a red-shifting effect in simulated UV-vis spectra. Removing the protons on the reduced rings also leads to a red shift but the effect is smaller. Spectral predictions for protonated states obtained by the computational methods used here were not accurate, although shifts were predicted. We are currently assessing the reasons for this and hope to be able to identify the factors involved. Regardless, protonation of **Ph_4_H_2_N_14_HEPT** provides an interesting route to affect tautomer identity or to vary the spectroscopic output of the molecules.

## Figures and Tables

**Table 1:** Bond-length averages for XRD and optimized structures.

Average Bond Length (Å)	X-ray Structure (*t*Bu_8_Ph_4_H_2_N_14_HEPT)	Optimized Structure (T0 (Ph_4_H_2_N_14_HEPT))
C-N	1.348	1.337
Acene C-C	1.439	1.448
Ph C-C	1.393	1.392
N-H	0.880	1.013

**Table 2:** TD-DFT results for Ox in gas phase. Results for compound T0 are given for comparison. The *f*-values are oscillator strengths.

Compound	HOMO-LUMO Gap (eV)	*λ*_abs_ (nm), *E*_abs_ (eV)	*f*_abs_	Transition	*λ*_em_ (nm), *E*_em_ (eV)	*f*_em_
**T0**	2.50	550 (2.25)	2.39	HOMO → LUMO(98%)	585 (2.12)	2.42
**Ox** (singlet)	1.81	657 (1.89)	2.23	HOMO-2 → LUMO(98%)		

**Table 3:** TD-DFT results for the anionic compounds in the gas phase. The results of compound **T0** are given for comparison. The *f*-values are oscillator strengths.

Compound	HOMO-LUMO Gap (eV)	*λ*_abs_ (nm), *E*_abs_ (eV)	*f*_abs_	Transition	*λ*_em_ (nm), *E*_em_ (eV)	*f*_em_
**T0**	2.50	550 (2.25)	2.39	HOMO → LUMO(98%)	585 (2.12)	2.42
**MA**	2.06	618 (2.01)	2.33	HOMO → LUMO(99%)	654 (1.90)	1.95
**DA**	1.75	664 (1.87)	2.73	HOMO → LUMO(100%)	695 (1.78)	1.83

**Table 4:** HOMO-LUMO gaps and relative energies of the four tautomers. Energies are relative to compound **T0**.

Compound	HOMO-LUMO Gap (eV)	Δ*E* (kcal/mol) ^a^	Δ*E*_0_ (kcal/mol) ^b^	Δ*G* (kcal/mol)
**T0**	2.499	0.000	0.000	0.000
**T1**	2.388	+2.592	+2.503	+2.454
**T2**	2.204	+11.566	+10.236	+13.409
**T3**	2.029	+31.629	+31.058	+30.512

^a^ Δ*E* is the total electronic energy. ^b^ Δ*E*_0_ is the zero-point corrected energy.

**Table 5:** TD-DFT results (gas phase) for the four tautomers studied. Oscillator strengths are given as *f* values.

Compound	*λ*_abs_ (nm), *E*_abs_ (eV)	*f*_abs_	Transition	*λ*_em_ (nm), *E*_em_ (eV)	*f*_em_
**T0**	550 (2.25)	2.39	HOMO → LUMO (98%)	585 (2.12)	2.42
**T1**	569 (2.18)	2.19	HOMO → LUMO (98%)	647 (1.92)	1.84
**T2**	606 (2.05)	2.07	HOMO → LUMO (98%)	690 (1.80)	1.75
**T3**	628 (1.97)	2.28	HOMO → LUMO (98%)		

**Table 6:** HOMO-LUMO gaps and relative energies of the eight studied protonated compounds. The compounds are arranged in series **T0**, **T1**, and **T2** shown in Figure 9, where the site of protonation is denoted by -**HX,** as also shown in Figure 9. Energies are relative to **T1**-**H2**.

Compound	HOMO-LUMO Gap (eV)	Δ*E* (kcal/mol) ^a^	Δ*E*_0_ (kcal/mol) ^b^	Δ*G* (kcal/mol)
**T0-H0**	2.355	+51.212	+50.571	+48.799
**T0-H1**	1.763	+17.952	+17.461	+17.195
**T0-H2**	1.565	+2.932	+2.908	+2.847
**T0-H3**	1.529	+4.485	+4.188	+4.518
**T1-H1**	1.531	+1.952	+1.906	+1.806
**T1-H2**	1.403	0.000	0.000	0.000
**T2-H1**	1.432	+4.242	+4.045	+3.988
**T2-H2**	1.349	+6.027	+5.753	+5.643

^a^ Δ*E* is the total electronic energy. ^b^ Δ*E*_0_ is the zero-point corrected energy.

**Table 7:** TD-DFT results for the eight studied protonated compounds in gas phase. The compounds are arranged in the series defined in Figure 9. The results of compound **T0** are given for comparison. The *f*-values are oscillator strengths.

Compound	HOMO-LUMO Gap (eV)	*λ*_abs_ (nm), *E*_abs_ (eV)	*f*_abs_	Transition	*λ*_em_ (nm), *E*_em_ (eV)	*f*_em_
**Tautomer-0 (Ph_4_N_14_Ph_4_H_2_)**	2.50	550 (2.25)	2.39	HOMO → LUMO (+98%)	585 (2.12)	2.42
**T0-H0**	2.36	Too unstable to be considered (+50 kcal.mol^−1^)				
**T0-H1**	1.76	777 (1.60)	1.40	HOMO → LUMO (100%)		
**T0-H2**	1.57	849 (1.46)	1.16	HOMO → LUMO (99%)		
**T0-H3**	1.53	863 (1.44)	0.98	HOMO → LUMO (99%)		
**T1-H1**	1.53	853 (1.45)	1.46	HOMO → LUMO (99%)	1002 (1.24)	1.35
**T1-H2**	1.40	907 (1.37)	1.27	HOMO → LUMO (99%)	1071 (1.16)	1.26
**T2-H1**	1.43	891 (1.39)	1.43	HOMO → LUMO (99%)		
**T2-H2**	1.35	932 (1.33)	1.28	HOMO → LUMO (99%)		

## Data Availability

Data are contained within the article and Supplementary Materials.

## Supplementary Materials

The following supporting information can be downloaded at https://www.mdpi.com/article/10.3390/molecules29102407/s1, Figure S1: Thermal ellipsoid diagram (50% probability level) of ***t*Bu_8_Ph_4_H_2_N_14_HEPT**; Figure S2: Calculated electronic absorption spectra for tautomer **T1** and the monoprotonated tautomers **T1-H1**, **T1-H2**, and **T1-H3**; Figure S3: Calculated electronic absorption spectra for tautomer **T2** and the monoprotonated tautomers **T2-H0**, **T2-H1**, **T2-H2**, and **T2-H3**. Coordinates of the calculated structures (.mol file) openable by the Mercury crystal structure visualization program (available free of charge at https://www.ccdc.cam.ac.uk/solutions/software/free-mercury; accessed on 15 May 2024).
