# Supplementary material for: Protic Processes in an Extended Pyrazinacene: The Case of Dihydrotetradecaazaheptacene
Source: Molecules. 2024 May 20;29(10):2407. doi: 10.3390/molecules29102407 (PMC11124472; doi:10.3390/molecules29102407)
Supplement: Supplementary file 1 [file molecules-29-02407-s001.zip › molecules-2996207-supplementary.pdf]

Supplementary Information

Protic Processes in an Extended Pyrazinacene: The Case of Dihydrotetradecaazaheptacene

Aël Cador, Samia Kahlal, Gary J. Richards, Jean-François Halet,* and Jonathan P. Hill*

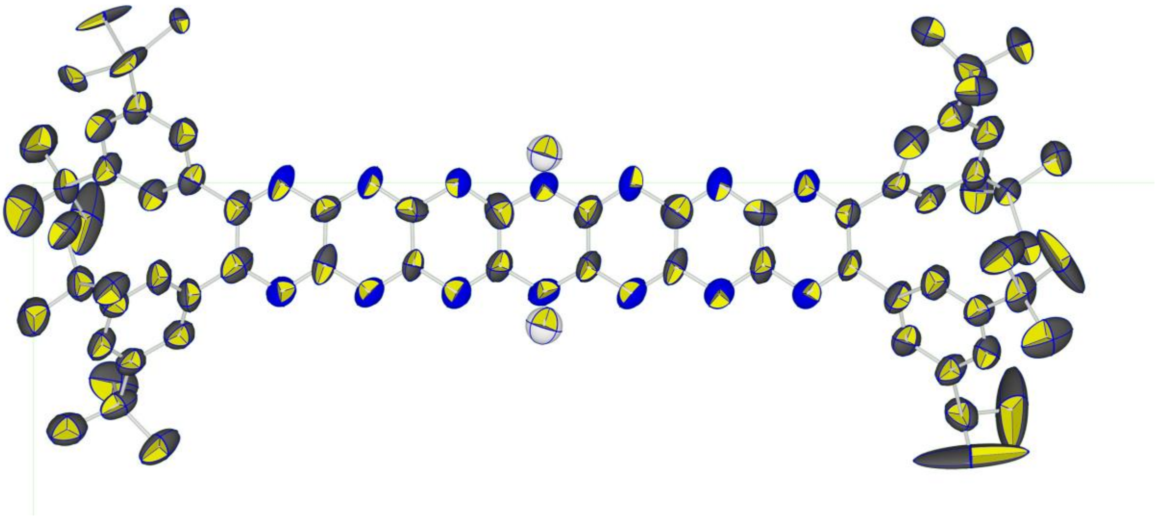

Figure S1. Thermal ellipsoid diagram (50 % probability level) of ***t*Bu$_8$Ph$_4$H$_2$N$_{14}$HEPT** reveals highly disordered t-butyl groups attached to a relatively well ordered N$_{14}$-pyrazinacene core.

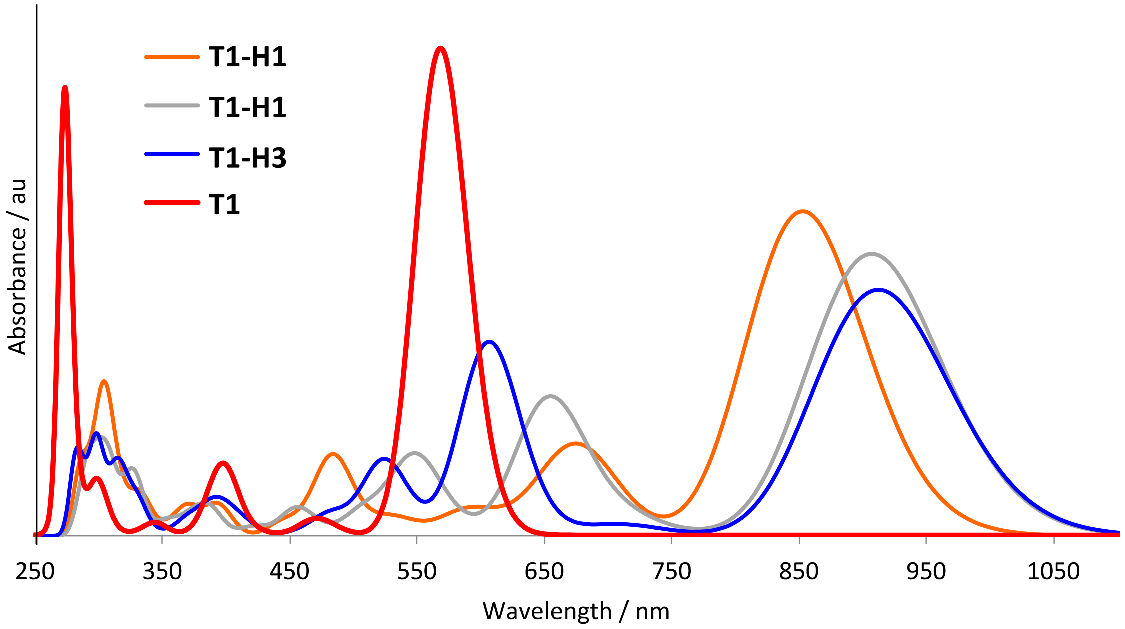

Figure S2. Calculated electronic absorption spectra for tautomer **T1** and the monoprotonated tautomers **T1-H1**, **T1-H2**, and **T1-H3**.

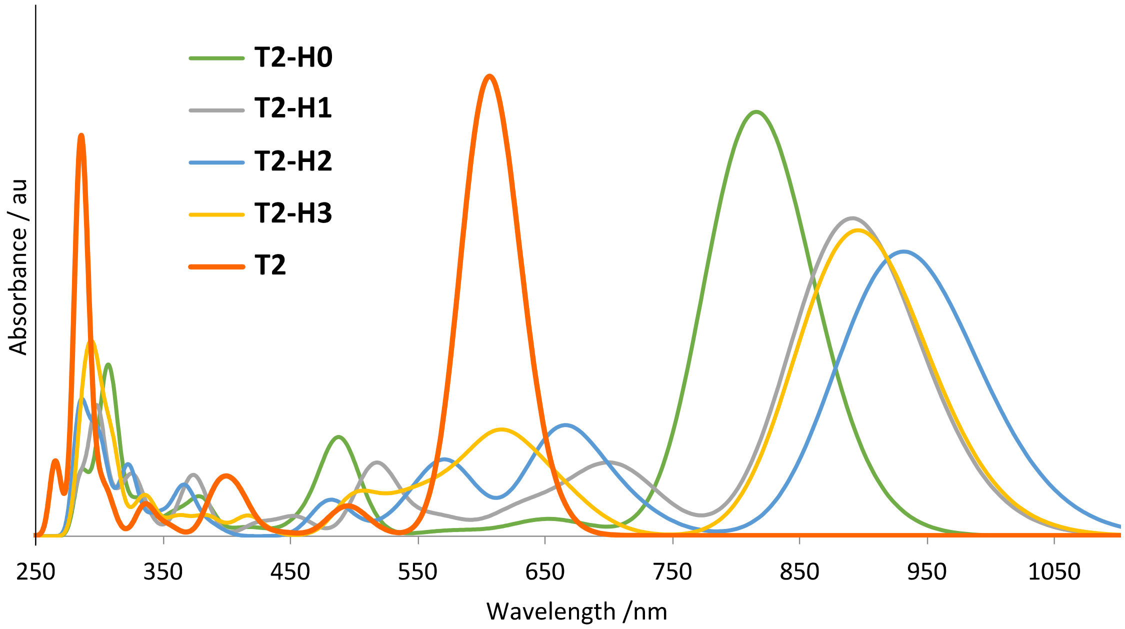

Figure S3. Calculated electronic absorption spectra for tautomer **T2** and the monoprotonated tautomers **T2-H0**, **T2-H1**, **T2-H2**, and **T2-H3**.
